# General Characteristics of patients with Primary Biliary Cholangitis from Türkiye and Denmark

**DOI:** 10.5152/tjg.2025.0201253

**Published:** 2025-04-02

**Authors:** Cumali Efe, Ersin Batıbay, Tugrul Purnak

**Affiliations:** 1 Department of Gastroenterology, Harran University Hospital, Şanlıurfa, Türkiye; 2 Department of Gastroenterology, Hepatology, and Nutrition, University of Texas Health Science Center at Houston McGovern Medical School, Texas, USA

Primary biliary cholangitis (PBC) is a rare, progressive, immune-mediated cholestatic liver disease that can progress to liver failure and death if left untreated. PBC primarily affects women, with a female-to-male ratio of 9:1.^1,2^ The diagnosis of PBC is based on the presence of cholestatic liver biochemistry, anti-mitochondrial antibodies (AMA), and liver histology findings.^2^ AMA are found in 90–95% of PBC patients and are highly disease-specific autoantibodies. A subgroup of anti-nuclear antibodies (ANA), such as ANA with a rim-like (gp210 protein) and multiple-dot (sp100 protein) indirect immunofluorescence staining pattern on HEp-2 cells, represent additional disease-specific autoantibodies.^3,4^

Currently, ursodeoxycholic acid (UDCA) is the first-line treatment for PBC, while obeticholic acid, elafibranor, seladelpar, and fibrates are second-line therapies for patients who do not respond to or cannot tolerate UDCA.^5,6^ Several UDCA biochemical response criteria (Barcelona, Paris I, Rotterdam, Toronto, Paris II, POISE) and risk scores (GLOBE and UK-PBC) have been developed to identify which groups of PBC patients are at risk of adverse outcomes.^7^

Fatigue and pruritus are debilitating PBC-specific symptoms. Managing these symptoms requires additional efforts, as neither UDCA nor second-line therapies are fully effective in controlling them.^1,2^

The incidence and prevalence of PBC vary significantly by ethnic population and geographical region. The highest incidence in Caucasian populations has been reported in Northern Europe, while the lowest incidence has been observed in the Indian subcontinent and Africa.^8^ A systematic review reported that the incidence of PBC varies from 0.33 to 5.8 per 100,000 per year, with prevalence rates ranging from 1.91 to 40.2 per 100,000.^9^ These variations suggest that genetic and environmental factors may influence PBC onset, but more real-world data are needed to confirm this hypothesis.

In this context, Eruzun et al^10^ evaluated the clinical phenotype and treatment response in two PBC populations from Denmark and Türkiye. The study findings provide valuable insights and help raise disease awareness in both countries. However, several aspects require further clarification.

Some clinical characteristics of Turkish and Danish PBC patients differed within the prevalent cohort. Danish patients were younger and had lower AMA positivity rates at the time of PBC diagnosis, while laboratory markers and cirrhosis frequency were similar in both populations. A large population-based study from the United Kingdom has convincingly shown that PBC in young women (<50 years) is less responsive to UDCA treatment.^11^ In contrast, the current study found that Turkish PBC patients were younger but had significantly higher UDCA response rates than Danish patients. These discrepant results warrant further discussion. Other factors, such as alcohol use, smoking, and concomitant steatosis-related liver disease, should also be considered, as they may influence treatment response.

In Danish and Turkish populations, AMA positivity was 75% vs. 92%, respectively. It is known that AMA presence or titer is not associated with prognosis or therapy response in PBC. ANA is detected in approximately 30-50% of PBC patients.^2,3^ Two types of ANA, distinct on indirect immunofluorescence, are called PBC-specific ANAs. The use of PBC-specific ANAs can reduce AMA-negative PBC cases to less than 5%.^3^ Unlike AMA, PBC-specific ANAs have prognostic value, as they can predict UDCA non-response and liver transplant-free survival.^1,2^ In this study, PBC-specific ANA status was not reported, even though these autoantibodies are increasingly recognized as diagnostic and clinical markers in PBC.

In the current study, the authors presented only UDCA response data from their population. A previous study showed that both UDCA response criteria and scoring systems effectively predicted outcomes in PBC cohorts from Türkiye and Denmark.^7^ However, UDCA non-response alone is no longer considered a sufficient surrogate criterion for predicting PBC outcomes, as several alternative therapies can induce biochemical responses in UDCA non-responders.^5,6^ This study did not provide data on second-line therapies, leaving uncertainty about available treatment options in these countries and how many UDCA non-responders achieved a biochemical response with additional therapies. To better define PBC outcomes, more reliable surrogates, such as liver-related mortality and liver transplantation rates, should be used.

It would also be valuable to assess the frequency of fatigue and pruritus in these PBC populations. A significant proportion of PBC patients experience pruritus and fatigue, both of which severely impact quality of life.^2^ Therefore, symptom assessment and management are critical strategies in PBC treatment. Increasing awareness and improving the management of PBC-related symptoms (fatigue and pruritus) in Türkiye and Denmark is essential.

In conclusion, this study highlighted geographic variations in clinical characteristics and UDCA response rates among PBC patients in Türkiye and Denmark. Further research is needed to identify potential individual and environmental factors that may influence PBC presentation and outcomes.

## Data Availability

The data that support the findings of this study are presented in the manuscript.

## References

[1] Ronca V Davies SP Oo YH Lleo A . The immunological landscape of primary biliary cholangitis: mechanisms and therapeutic prospects. Hepatology. Published online January 3, 2025. 2025. (10.1097/HEP.0000000000001225) 39774114

[2] Lindor KD Bowlus CL Boyer J Levy C Mayo M . Primary biliary cholangitis: 2018 practice guidance from the American Association for the Study of Liver Diseases. Hepatology. 2019;69(1):394 419. (10.1002/hep.30145) 30070375

[3] Ozaslan E Efe C Gokbulut Ozaslan N . The diagnosis of antimitochondrial antibody-negative primary biliary cholangitis. Clin Res Hepatol Gastroenterol. 2016;40(5):553 561. (10.1016/j.clinre.2016.06.001) 27567165

[4] Ergenc I Gozaydinoglu B Keklikkiran C Yilmaz Y . The risk of development of primary biliary cholangitis among incidental antimitochondrial M2 antibody-positive patients. Hepatol Forum. 2023;4(2):69 73. (10.14744/hf.2023.2023.0016) 37250930 PMC10209978

[5] Levy C Bowlus CL . Primary biliary cholangitis: personalizing second-line therapies. Hepatology. Published online November 12, 2024. 2024. (10.1097/HEP.0000000000001166) 39707635

[6] Barba Bernal R Ferrigno B Medina Morales E , et al. Management of primary biliary cholangitis: current treatment and future perspectives. Turk J Gastroenterol. 2023;34(2):89 100. (10.5152/tjg.2023.22239) 36843300 PMC10081121

[7] Efe C Taşçilar K Henriksson I , et al. Validation of risk scoring systems in ursodeoxycholic acid-treated patients with primary biliary cholangitis. Am J Gastroenterol. 2019;114(7):1101 1108. (10.14309/ajg.0000000000000290) 31241547

[8] Metcalf J James O . The geoepidemiology of primary biliary cirrhosis. Semin Liver Dis. 1997;17(1):13 22. (10.1055/s-2007-1007179) 9089907

[9] Boonstra K Beuers U Ponsioen CY . Epidemiology of primary sclerosing cholangitis and primary biliary cirrhosis: a systematic review. J Hepatol. 2012;56(5):1181 1188. (10.1016/j.jhep.2011.10.025) 22245904

[10] Eruzun H Bossen L Turan Gökçe D , et al. Clinical and biochemical characteristics of a Danish and Turkish cohort of incident and prevalent patients with primary biliary cholangitis. Turk J Gastroenterol. Publised online January 6, 2025. 2025. (10.5152/tjg.2025.24300) PMC12001446 40181751

[11] Carbone M Mells GF Pells G , et al. Sex and age are determinants of the clinical phenotype of primary biliary cirrhosis and response to ursodeoxycholic acid. Gastroenterology. 2013;144(3):560 569. (10.1053/j.gastro.2012.12.005) 23246637

